# Sublingual microrobotic pills for rapid and efficient drug delivery†

**DOI:** 10.1039/d5na00313j

**Published:** 2025-06-23

**Authors:** Nelly Askarinam, Chuanrui Chen, Vivian Vo, Michael Casares, Kyra Wu, Ethan Shen, Majd Iskandarani, Víctor de la Asunción-Nadal, An-Yi Chang, Tomohiro Yamamoto, Janna Sofia Sage-Sepulveda, Baha Öndeş, Zhenning Zhou, Zike Yan, Jesse Qualliotine, Joseph Wang

**Affiliations:** a Aiiso Yufeng Li Family Department of Chemical and Nano Engineering, University of California San Diego La Jolla California 92093 USA josephwang@ucsd.edu; b Moores Cancer Center, University of California San Diego La Jolla CA USA jqualliotine@health.ucsd.edu; c Department of Otolaryngology – Head and Neck Surgery, University of California San Diego La Jolla CA USA; d University of California San Diego, School of Medicine La Jolla California 92093 USA

## Abstract

Rapid drug delivery is critical in emergency medical circumstances where delays can result in adverse or life-threatening outcomes. The sublingual route holds significant promise for the swift systemic delivery of drugs, but is limited by slow passive diffusion mechanisms that hinder efficient drug transport. To address these challenges, we introduce here a novel sublingual microrobotic pill platform designed for rapid and efficient drug delivery. Our system incorporates magnesium-based microstirrers that accelerate pill disintegration and drug release *via* an active propulsion mechanism. Such bubble-propelled microstirrers generate strong hydrodynamic flows, enhancing local mixing and drug transport, allowing them to overcome the limitations of traditional diffusion-dominated delivery systems. Optimized through *in vitro* studies, this platform demonstrated robust motion capabilities in complex human saliva and accelerated drug release kinetics. In a rabbit model, using epinephrine as the model drug, the sublingual microrobotic pill achieved significantly faster drug delivery speed, efficiency, and bioavailability compared to a conventional pill. Moreover, when compared against the gold standard intramuscular injections, the microstirring pill provides competitive delivery speed and enhanced absorption profile, demonstrating its potential for use in the treatment of conditions like anaphylactic shock. Such user-friendly, non-invasive sublingual microrobotic pills can be readily employed for delivering a wide range of drugs, offering a versatile solution for acute conditions requiring rapid therapeutic onset or when enteral absorption is not feasible.

## Abstract 2

A sublingual microrobotic pill embedded with magnesium microstirrers that accelerates drug release and transport by active propulsion mechanism, enhancing bioavailability.

## Graphical abstract

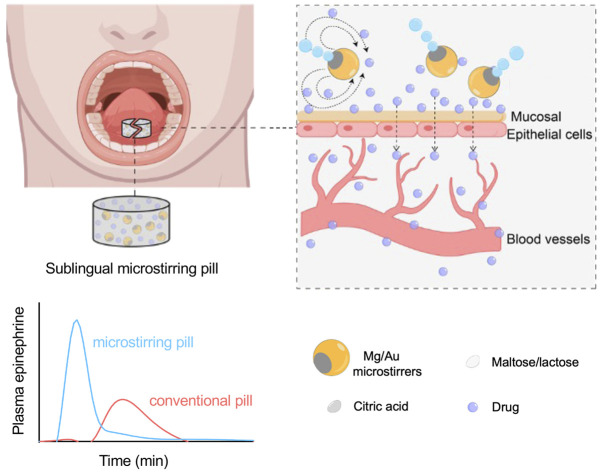

## Introduction

Drugs used in emergency situations demand rapid therapeutic action, as any delays can have life-threatening consequences.^1^ While oral delivery has long been the cornerstone of pharmaceutical therapeutics, its reliance on absorption pathways through the gastrointestinal (GI) tract significantly delays the onset of therapeutic effects, rendering it inadequate for urgent and acute emergency situations.^2^ Although intramuscular and intravenous injections address these limitations with faster therapeutic onset, they are invasive, require trained personnel or expensive auto-injectors, and are not universally accessible.^3,4^

Amid the growing need for non-invasive, patient accessible therapeutic options, the sublingual route emerges as a promising avenue for rapid delivery where drugs have a direct access point for systemic uptake through the rich vascular network beneath the tongue.^5–7^ Bypassing the hepatic first pass metabolism allows faster therapeutic onset, making it well-suited for emergency interventions.^8,9^ Existing sublingual formulations, such as nitroglycerin for angina, naloxone for opioid dependence, and edaravone dexborneol for stroke, have demonstrated the utility of this delivery route in acute conditions in which rapid onset is desired.^10–12^ However, the delivery efficiency of current sublingual platforms is limited by poor hydrodynamics and their reliance on passive absorption mechanisms.^7^ Thus, the lack of an active delivery mechanism impedes effective drug transport across the sublingual mucosal barrier.

To address this challenge, we propose a sublingual microrobotic pill platform designed to address acute conditions where rapid and efficient drug delivery remain vital. Microrobotic technology offers a transformative solution in enhancing drug delivery efficiency by leveraging an active propulsion mechanism whereby microrobots can enhance local drug release, mixing and transport, while overcoming diffusion barriers.^13,14^ Various microrobotics platforms have been developed, powered by external fields (magnetic, electric, acoustic), chemical reactions, or natural self-propulsion mechanisms.^15–19^ Such technologies have shown promise in delivering therapeutics to specific regions of the body, addressing challenges such as short retention times and low bioavailability.^20,21^ A particularly promising approach involves integrating microrobots directly into pharmaceutical pills, significantly enhancing drug delivery efficiency and bioavailability, specifically for GI-based systems.^22–24^ One attractive robotic pill employs microstirrers composed of 20 μm magnesium (Mg) Janus microparticles with an asymmetric outer inert coating.^25^ The exposed Mg layer provides a localized region by which these active microrobots can react with their surrounding media and propel *via* a stream of hydrogen gas microbubbles, providing an efficient *in situ* stirring effect, which improves drug release, distribution, and absorption. However, while prior efforts have demonstrated their superior performance in enteral delivery systems, the potential of this platform for sublingual delivery especially in acute, time-sensitive circumstances remain largely unexplored.

Herein, we demonstrate the first sublingual microrobotic pill platform. Targeting acute conditions where drug delivery speed and efficiency remain paramount, the composition of the sublingual microrobotic pill was carefully designed and optimized *in vitro* to operate effectively in both artificial and the complex milieu of human saliva. Traditional microrobotic pill platforms deliver their pharmaceutical payload within the stomach or GI tract, where an acidic environment activates robotic propulsion. The conditions in the oral cavity, however, are vastly different. Furthermore, oral cavity mucosa cannot tolerate highly acidic conditions for extended periods of time.^26^ Thus, our sublingual platform was deliberately engineered to coencapsulate microstirrers with citric acid, a commonly used food additive and flavour enhancer,^27^ to provide the necessary acidic conditions for these microstirrers to operate safely, swiftly and effectively in the oral cavity, a novel delivery route for the microrobot.

**Fig. 1:**
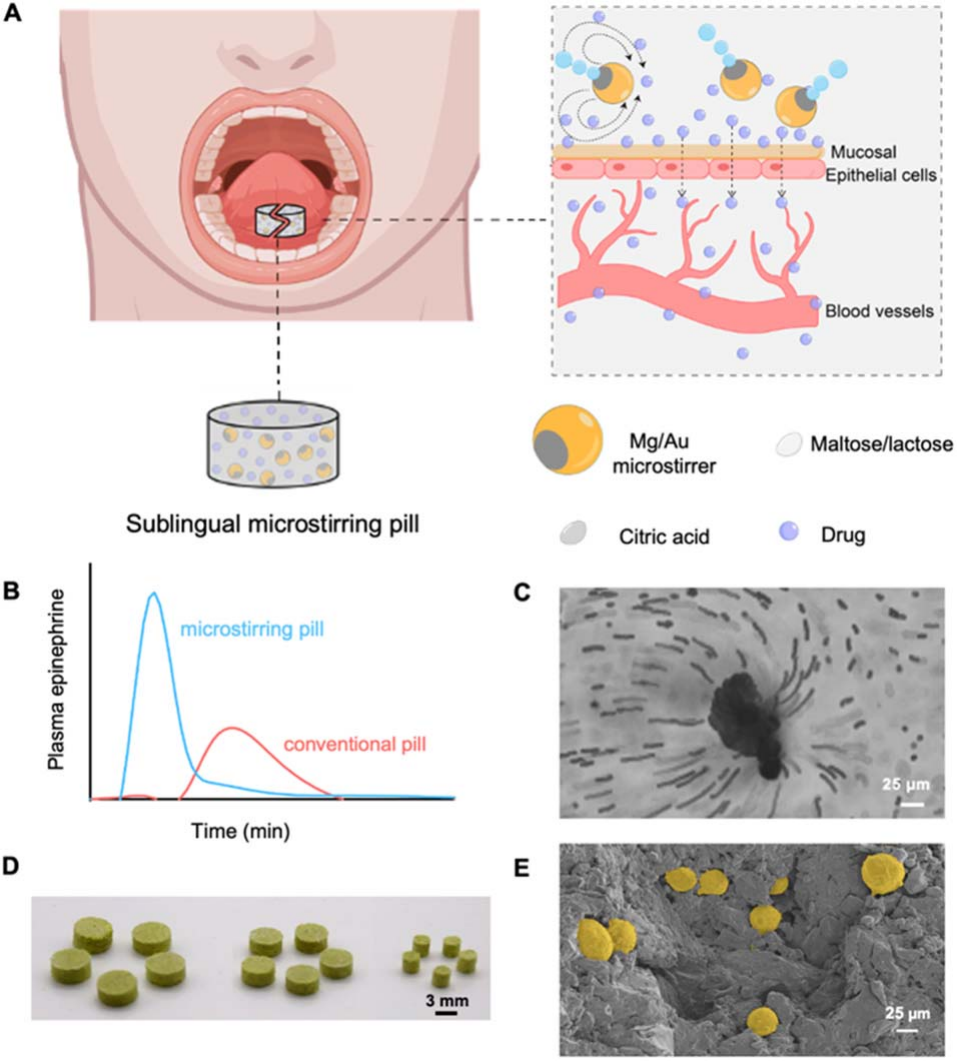
Schematic of the sublingual microstirring pill platform. (A) Illustration of the Mg/Au microstirring pill administered sublingually and the motion of Mg/Au microstirrers with enhanced drug delivery and absorption across the oral mucosal lining. (B) Representative resulting peaks in plasma levels obtained from a model sublingual epinephrine microstirring *vs.* conventional pill. (C) Hydrodynamic flow surrounding a Mg/Au microstirrer immersed in artificial saliva; polystyrene tracer microparticles (2 μm) were used to visualize local fluid mixing. (D) Photograph image of sublingual microstirring pills with different diameters (3, 7 and 8 mm) dyed with yellow food coloring. (E) SEM image of the cross-section of a sublingual microstirring pill with embedded Mg/Au microstirrers pseudo-dyed in gold.

Upon sublingual administration, rapid pill disintegration and dissolution begins: the presence of hydrogen ions from the citric acid produces active movement of the released microstirrers, thereby generating a robust hydrodynamic flow while enhancing local fluid mixing and drug transport (Fig. 1A–C). *In vitro* testing revealed accelerated release kinetics of the microstirring pill compared to conventional pills in two model drugs: epinephrine and propranolol. An *in vivo* pharmacokinetic study using a rabbit model examined the potential of utilizing the robotic sublingual pill for enhancing the delivery of epinephrine, the most vital drug in the treatment of anaphylactic shock, an acute, life-threatening allergic reaction requiring immediate therapeutic intervention.^28^ Our microstirring epinephrine pill demonstrated superior delivery speed and bioavailability compared to a passive conventional pill, reflecting the enhanced transport across the mucosal lining (Fig. 1A). Furthermore, when compared against an intramuscular injection of epinephrine, the current gold standard treatment for anaphylaxis, our microstirring sublingual pill achieves a competitive delivery speed with enhanced systemic epinephrine absorption, highlighting its potential to serve as an innovative tool in acute medical conditions.

Beyond its high drug delivery efficiency, the sublingual microrobotic pill offers practical advantages: it is user-friendly, non-invasive and cost-effective. This versatile platform is adaptable for delivering a variety of drugs, expanding its applications to both medical emergencies and non-emergency cases where rapid therapeutic action is desired. By combining efficiency, accessibility, and versatility, the sublingual microrobotic pill represents a transformative approach to modern drug delivery, poised to address unmet needs across diverse patient populations.

## Experimental

### Microstirrer and pill fabrication

Mg/Au microstirrers were fabricated using commercial ∼20 μm Mg microparticles (TangShan WeiHao Magnesium Powder Co.) as the core. The particles were dispersed onto glass slides previously coated with a 0.1 mL of 0.5 wt% polyvinylpyrrolidone ethanolic solution (Spectrum Chemical MGF CORP). The dispersed Mg particles were later sputtered with a thin layer of Au using a Denton Discovery 18 Sputtering System for 3 min, a DC power of 200 W, argon flow of 2.4 mT and no rotational speed to fabricate the Mg/Au microstirrers. Microstirrers were detached from the glass slides by scratching off with a blade.

To study the drug release profile of epinephrine and propranolol sublingual microstirring pills with various microstirring loading amounts (1.5, 2.5 and 5 wt%), the pills were prepared with 3% drug, 75% citric acid (Fischer Scientific) and a remaining wt% consisting of a mixture of lactose (Spectrum Chemical MGF Corp.) and maltose (Spectrum Chemical MGF Corp.) (60 : 40 w/w). First, epinephrine bitartrate (Thermo Scientific Chemicals) or propranolol hydrochloride (Sigma-Aldrich) was triturated along with maltose, lactose and citric acid (Fischer Scientific) using a mortar and pestle. Once the mixture was homogenized, microstirrers were incorporated and lightly mixed in the mixture. Precise control of the microstirrer loading and post-fabrication verification of the total weight of each pill was used to confirm consistency. A separate control pill group was prepared without the addition of microstirrers. Subsequently, the pill mixture was transferred to a 2-part pill mould. The mixture was filled in the cavities of a stainless-steel pill mould plate by compression, by applying pressure using a PEG plate to ensure tight packing (Fig. S1†). Once the cavities of the pill mould were filled, a couple of drops of anhydrous ethanol was added to either side of the pill mould to help bind the pill matrix powder, then dried with an air gun and subsequently lowered onto the PEG plate to eject the dried pills from the pill mould. The freed pills were dried overnight at 25 °C in a vacuum pump and later sealed and stored at 4 °C until further use. To prepare epinephrine or propranolol microstirring pills with varying drug concentrations (3, 10, 20 wt%), the maltose–lactose mixture was adjusted to account for the changing drug concentration while holding the citric acid and microstirrer weight constant at 75% and 2.5%. To prepare the microstirring pills without any drug loading, the same method was used, but with pill compositions adjusted such that an increasing wt% of maltose–lactose mixture was applied to compensate for the replaced drug mass. The drug remained stable during the fabrication and storage, and no adverse interactions were observed between the drug and excipients (such as lactose, maltose, or the microstirrers).

### Characterization of microstirrer and microstirring pill

Scanning electron microscopy (SEM) images were taken for a single microstirrer and cross-section of a microstirring pill conducted with a FEI Apreo instrument, using an acceleration voltage of 15 and 3 kV, respectively. Samples were pre-sputtered with an iridium coating before visualization. Energy dispersive X-ray analysis (EDX) mapping analysis was performed over the area of a single microstirrer to determine the presence of Mg and Au using an Oxford EDX detector attached to the SEM instrument and performed by Pathfinder software.

### Microstirrer motion test

Artificial saliva was prepared based on a previous report.^29^ Mg/Au microstirrers were immersed in artificial saliva and their motion was captured on video using an inverted Nikon microscope coupled with a CCD, *n* = 5. Static Mg/Au microstirrers were fabricated by incubating Mg/Au microstirrers in water at 80 °C for 72 hours. Human saliva was collected from three healthy subjects. All media was supported with 0.5 wt% Triton X-100. For the hydrodynamic studies, 2 μm polystyrene (PS) particles were used as tracers, videos were later analysed by ImageJ.

The particle velocimetry images were obtained by tracking the position of tracer particles around a single microstirrer and a non-motile control attached to the surface of a glass slide. Videos were analysed using the TrackMate plugin in ImageJ. The position of the tracer particles was recorded and a custom Python script was used to estimate the local cumulative speed by dividing each frame with a 200 × 200 mesh and assigning a value to each point according to the velocity of nanoparticles normalized with the distance to each point of the mesh. The resulting motion maps were summed to show the cumulative motion, normalized with time and smoothed by applying a Gaussian averaging function.

### *In vitro* epinephrine and propranolol release studies

The drug loading per pill was set at 3, 10, or 20 wt% of the total pill mass (26 mg) for both epinephrine and propranolol. Each pill was placed in a 15 mL scintillation vial containing 1.5 mL of artificial saliva and incubated at 37 °C. At each time point, 200 μL aliquots were taken and replenished with fresh artificial saliva. Absorbance values at each time point were measured to quantify epinephrine at 279 nm using UV-2450 (Shimadzu Corporation) and propranolol at 290 nm using a BioTek Synergy Mx microplate reader in accordance with the manufacturer's instructions.

### *In vivo* pharmacokinetic epinephrine studies

6-Month-old female New Zealand White rabbits (*n* = 3) were used in this study. All animal procedures were approved by the Institutional Animal Care and Use Committee of UCSD (protocol #S24003). The rabbits were housed under standard laboratory conditions with controlled temperature, humidity, and a 12-h light/dark cycle. The rabbits were anesthetized and administered an epinephrine microstirring or control pill (3 × 6 mm) loaded with 10 mg epinephrine in the sublingual space on the underside of the tongue or provided an IM epinephrine injection (0.3 mg) in the anterior thigh. The sublingual dose of 10 mg was selected to ensure sufficient drug delivery for systemic uptake based on previous studies using higher doses (*e.g.*, 30 mg) with passive sublingual systems, while the 0.3 mg IM dose reflects the standard clinical adult dose.^28^ 0.5 mL of water was co-administered in the oral cavity to assist with pill dissolution. Five minutes after pill administration, any remaining pill was removed, and 25 mL of water was introduced into the rabbit's mouth to wash away any remaining epinephrine in order to reduce the risk of harmful local tissue vasoconstriction caused by epinephrine. At each time point, 200 μL of whole blood was collected from the central auricular artery, and plasma epinephrine levels were quantified using a commercial ELISA kit (Abnova K1882) and glucose measurements made using an ACCU-CHEK Aviva Plus Glucometer. All epinephrine values were normalized from baseline (*t* = 0). Statistical analyses were performed on GraphPad Prism 10, using one-way ANOVA and Tukey's multiple comparisons post-tests to determine statistical difference in *C*_max_, *T*_max_ & AUC.

It should be noted that, in our *in vivo* experiments with the post-administration oral cavity washing steps in place, one rabbit provided a control pill developed a self-limited oral ulcer, likely due to the local tissue vasoconstriction effect of epinephrine. The symptoms were present for three days before resolving spontaneously. Similarly, in the IM injection group, one rabbit exhibited minor ulcers, which resolved later with minor medical intervention.

## Results and discussion

Fig. 1A illustrates a schematic representation of the sublingual microrobotic pill on the underside of the tongue, a vascular-rich entry point facilitating rapid systemic drug absorption, bypassing delays associated with first-pass metabolism in traditional oral delivery. The sublingual microstirring pill matrix comprises Mg/Au microstirrers, maltose, lactose, and citric acid. The microstirrers consist of Mg microparticles asymmetrically coated with a thin outer Au layer, leaving a small area of Mg core exposed. This exposed core reacts with the surrounding saliva in the presence of citric acid, establishing a localized acidic environment necessary for activating microstirrer motion. This produces a stream of H_2_ gas microbubbles that propel the stirrers and induce localized stirring, accelerating pill disintegration and drug release. Fig. S2† displays the characterization of a Mg/Au microstirrer with an exposed Mg inner core (shown in left) and corresponding elemental mappings of Mg and Au (in middle and right) using SEM with energy-dispersive X-ray spectroscopy.

The robust hydrodynamic mixing provided by the microstirrers allows for rapid and efficient transport of the therapeutic payload in the media (taken from ESI Video 1†), enhancing absorption provided by the increased concentration of drug brought near the mucosa surface. This results in a swift increase in plasma concentration of a model drug (*i.e.* epinephrine) in a microstirring pill compared to a conventional pill without stirrers (Fig. 1B). The enhanced hydrodynamic mixing caused by the microstirrers was evaluated using passive PS microparticle tracers (2 μm) depicted in Fig. 1C.

To showcase the versatility and scalability of our pill platform, we fabricated multiple sublingual pills with various diameters (3, 7 and 8 mm) shown in Fig. 1D, and provide a cross-sectional view of the microstirring pill with embedded Mg/Au microstirrers pseudo-coloured in gold displayed in Fig. 1E.

**Fig. 2:**
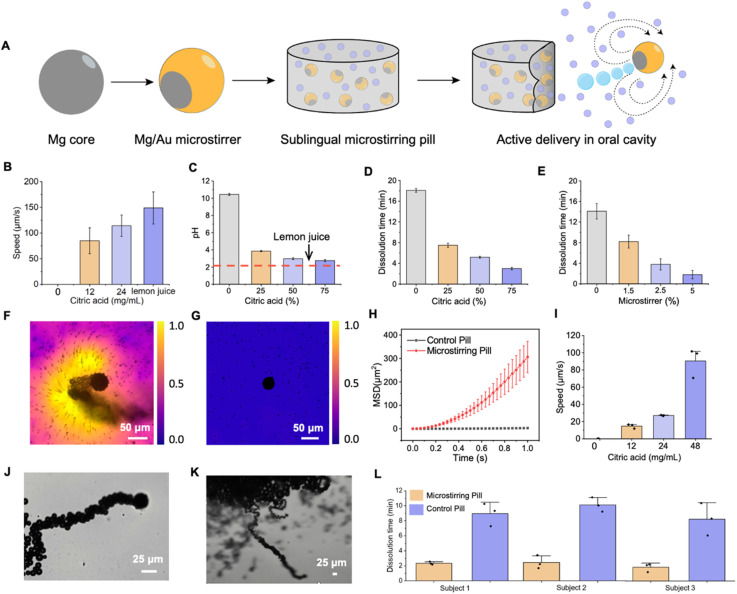
Characterization and optimization of the sublingual microstirrer pill platform. (A) Schematic illustration of the design and fabrication of sublingual microstirrer pill composed of Mg/Au microstirrers containing an inner Mg core and outer Au shell encased in a pill matrix. Upon activation, the microstirring pill disintegrates releasing the microstirrers, enhancing hydrodynamic flow and accelerating drug release. (B) Speed of free Mg/Au microstirrers in artificial saliva supplemented with 0–24 mg mL^−1^ of citric acid *vs.* lemon juice. (C) pH study of dissolved sublingual microstirrer pill (2.5% microstirrers) with varying citric acid compositions (0–75%) in 1.5 mL artificial saliva *vs.* lemon juice. (D) Dissolution study of sublingual microstirring pills with varying citric acid levels (0–75%) and 2.5% microstirrers. (E) Dissolution study of sublingual microstirring pills (75% citric acid) with varying microstirrer concentration (0–5%). (F) Microscope images and speed mapping demonstrating enhanced fluid mixing with a Mg/Au microstirrer compared to (G) static Mg/Au microstirrer in artificial saliva with 2 μm PS particles as tracers. (H) Mean-squared displacement (MSD) of tracer particles with a Mg/Au microstirrer and static Mg/Au microstirrer in 1 s duration. (I) Speed of microstirrers in human saliva supplemented with varying citric acid concentrations. Microscope images of free microstirrer moving in human saliva (J) and embedded microstirrers released from motor pill (K) moving in human saliva. (L) Dissolution time of sublingual microstirring and control pills in human saliva across different subjects at body temperature.

Fig. 2 depicts the characterization and optimization of the sublingual microstirrer pill platform. In Fig. 2A, 20 μm Mg spheres serve as the core of the microstirrer, coated asymmetrically with gold to form the Janus structure. Upon exposure to surrounding fluid, the pill rapidly disintegrates, enhancing local hydrodynamics and enabling efficient drug distribution, supporting rapid drug release and transport across the mucosal lining and into the bloodstream. While Mg microstirrers have demonstrated propulsion in biologically relevant media, including gastric and intestinal fluids, their activity in saliva remains largely unexplored. Conditions in the oral cavity that necessitate microstirrer motion require a rich supply of hydrogen ions to active their propulsion and operate swiftly in the oral cavity. However, the highly acidic conditions found in gastric fluid may pose harm to the oral mucosal lining.^26^ Thus, citric acid, a widely used food additive and flavor enhancer, was first investigated for its potential role in activating microstirrer propulsion. In the presence of citric acid, protons (H^+^) are readily available to react with the exposed magnesium surface of the microstirrers, initiating a redox reaction:Mg(s) + 2H^+^(aq) → Mg^2+^(aq) + H_2_(g)

Fig. 2B highlights the motion of microstirrers in artificial saliva supplemented with citric acid. When no citric acid was added, the Mg/Au microstirrers remain static. However, upon increasing the citric acid concentration from 0 to 24 mg mL^−1^, their speed rose as high as 114 μm s^−1^. A higher concentration of hydrogen ions present in the media increased the interaction between the ions and the exposed Mg surface, enhancing the rate of hydrogen gas evolution and resulting in accelerated speed of the microstirrers.

To ensure the safety of the acidity level provided by the citric acid excipient, we prepared microstirring pills with varying citric acid levels and measured the pH after complete dissolution (Fig. 2C). Pills were dissolved in 1.5 mL of artificial saliva to stimulate typical oral cavity conditions.^30^ While the pH of the dissolved microstirring pill decreased with increasing citric acid content, its acidity level never surpassed that of pure lemon juice when prepared with up to 75% citric acid. This suggests that our microstirring pill has clinically tolerable pH levels. In contrast, microstirring pills without citric acid exhibited higher alkalinity, attributed to the dissociation of hydroxide ions from the magnesium hydroxide product, a result of the reaction between the exposed Mg surface and the surrounding aqueous environment.

Next, we studied whether our microstirring pills provided a competitive dissolution profile, a crucial time-sensitive prerequisite necessary for achieving timely and rapid drug absorption. We investigated the dissolution profiles of the microstirrer pills prepared with varying citric acid and microstirrer concentrations in artificial saliva. As shown in Fig. 2D, pills without citric acid dissolved in ∼18 min, while those with 75% citric acid dissolved in just under 3 min (with 2.5% microstirrer loading). A series of representative images illustrating the time-dependent dissolution process is presented in Fig. S3.† The low dissolution rate of the microstirring pills without citric acid is in part due to its reliance on a passive mechanism for pill disintegration and subsequent solubilization due to microstirrer inactivity along with the low volume of artificial saliva present, typical of oral cavity conditions. In contrast, the microstirring pill relies on an active mechanism where the activated microstirrers in the presence of citric acid amplify Mg-surface reaction, elevating microbubble generation and accelerating pill disintegration. Similarly, when exploring the effect of increasing microstirrer concentration from 0 to 5% on pill dissolution time, a notable decline in dissolution time from 14.1 to 1.8 min is observed, further highlighting the critical role of microstirrer activity on pill's disintegration performance (Fig. 2E).

To investigate the mechanism behind the microstirrer pill's enhanced dissolution and potential in improving drug transport, we analysed their hydrodynamic flow in artificial saliva. Efficient drug delivery speed depends on rapid pill dissolution and drug transport, which is heavily influenced by the mixing dynamics surrounding the pill. Microbubbles generated by the Mg-acid reaction were released at a frequency of ∼25 Hz and created a robust dynamic fluid mixing effect as illustrated by tracer particles trajectories and tracer speed mapping shown in Fig. 2F. In contrast, static Mg/Au microstirrer exhibited limited hydrodynamics, minimal mixing, and negligible tracer particle speed emphasizing the importance of the active motion of microstirrers (Fig. 2G). Moreover, to quantify the hydrodynamic advantage of the microstirrers pills, we measured the mean squared displacement (MSD) of the tracer particles incubated with the Mg/Au microstirrer compared to a static Mg/Au microstirrer shown in Fig. 2H (ESI Video 1†). The MSD of the active microstirrer demonstrated exponential growth across time, indicating dominant convective mixing, whereas the static microstirrer exhibited only Brownian motion, indicated by a linear line with low MSD. This quantitative analysis shows the capacity of microstirrers to generate significant fluid motion, directly contributing to faster dissolution rates and drug transport, critical for enhancing drug absorption.

Recognizing that artificial saliva may not fully mimic the complexities of real biological environments, we also evaluated the performance of the Mg/Au microstirrers in human saliva, as shown in Fig. 2I–K. This step was crucial for verifying that the microstirrers could maintain their motion performance in a natural, protein-rich fluid where factors like viscosity and fouling may pose challenges in motion ability of small-scale microstirrers. Remarkably, the motion speed of the Mg/Au microstirrers in human saliva was comparable to the speed of free microstirrers in artificial saliva, indicating negligible effects from these factors (ESI Video 2†). The motion trajectories of free Mg/Au microstirrers and those released from pills in human saliva were similar, as shown in Fig. 2J and K, demonstrating that their motion remained unaffected even after incorporated into a pill (ESI Video 3†).

Finally, to assess the real-world applicability of the microstirring pill, we studied its dissolution time in saliva collected from three human subjects. This accounted for individual variability in saliva composition and viscosity, which are critical factors in practical sublingual drug delivery scenarios.^31^ Differences in pH and protein content in saliva may affect the rate of the Mg-acid reaction, which drives the microstirrer activity and subsequent dissolution. Similarly, variations in saliva viscosity could alter the hydrodynamic conditions and the efficiency of drug mixing and transport.

Despite these differences, the microstirrer pill exhibited significantly faster dissolution compared to the control pills across all three subjects, highlighting its potential as a platform for rapid and active drug delivery. These results demonstrate the platform's capability to function effectively in unprocessed human biological samples, offering distinct advantages especially in situations requiring immediate therapeutic action.

**Fig. 3:**
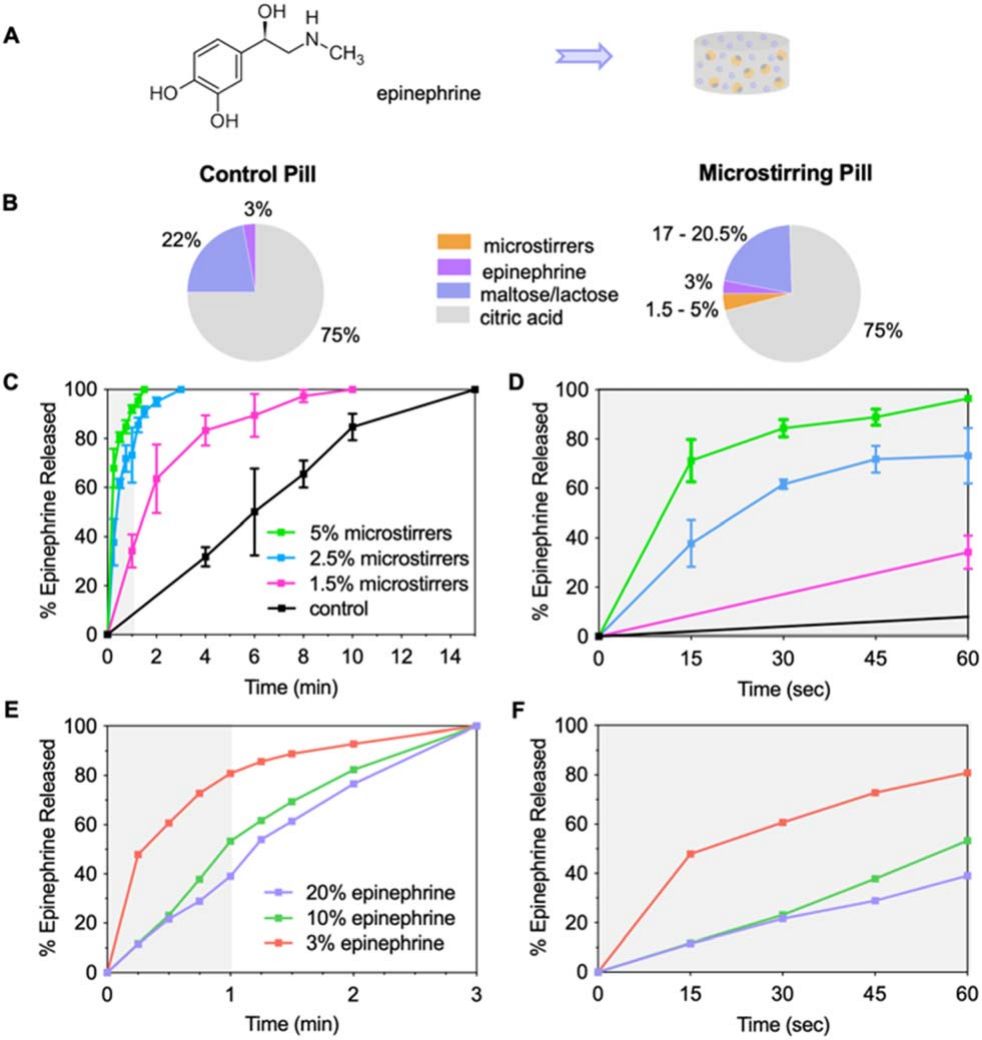
*In vitro* release kinetics of epinephrine from sublingual microstirring and control pills. (A) The chemical structure of epinephrine with side-by-side illustration of a microstirring pill. (B) Pie chart illustrating pill compositions (%) of control and microstirring pills with various microstirring loading amounts. (C) Kinetic study of epinephrine release from microstirring pills with varying loading amounts of microstirrers *vs.* control pill across 15 min and (D) at first min mark with 3% epinephrine loading. (E) Kinetic study of epinephrine release from microstirrer pills with various loading amounts of epinephrine and 2.5% microstirrer across 3 min and (F) at the first min mark.

Next, to determine how the enhanced dissolution rate of microstirring pill affects the drug delivery behaviour, we subsequently studied the *in vitro* release kinetics of epinephrine as the model drug due to its crucial clinical importance in requiring rapid and efficient delivery to the bloodstream to treat anaphylactic shock. Microstirring pills with varying microstirrer compositions (1.5–5 wt%) were prepared along with a control pill group with no microstirrers. The schematic and composition of the microrobotics pill are illustrated in Fig. 3A and B. The pill matrix was formulated using a mixture of epinephrine, maltose, lactose and citric acid, the latter providing the necessary acidic conditions to activate microstirrer motion, as discussed previously. Each pill was dissolved in 1.5 mL of artificial saliva at 37 °C. Aliquots were collected at various time points until full pill dissolution was achieved and epinephrine was subsequently quantified immediately thereafter using UV-vis spectroscopy (Fig. S4†).

As shown in Fig. 3C and D, the control pill exhibited a slow gradual release profile, with complete release occurring at ∼15 min. In contrast, the microstirring pill, demonstrated a significantly faster release of epinephrine, achieving complete release as early as 1.5 min with 5% (wt%) microstirring loading and 3 min with 2.5% microstirring loading. More significantly, within the first min mark, 5% and 2.5% microstirring pills demonstrated 92% and 73% release as opposed to only 8% in controls with 12- and 9.5-fold higher release respectively. This elucidates the superior drug release rate of epinephrine from the microstirring pill whereby the microstirrer activity not only accelerated the pill's dissolution but also facilitated rapid drug diffusion. When increasing the epinephrine loading by ∼3 and 6.5 times in the microstirring pill, full dissolution rate was not severely impacted, but rather an initial drop in release behaviour was observed by 1.5 and 2 folds respectively at the first min mark (Fig. 3E and F).

A second drug model utilizing propranolol was studied to further demonstrate the versatility of the microrobotic pill platform. Propranolol, a beta-blocker commonly used in the treatment of hypertension and arrhythmias, was selected due to its need for rapid drug delivery in various clinical conditions.^32^ As shown in Fig. S5A,† the control pill exhibited a slow-release profile of propranolol across 15 min. Much like the epinephrine control pill, this was largely due to its inherent dependence solely on passive diffusion accompanied with a low volume of artificial saliva present for dissolution and subsequent drug release, mimicking the conditions of the oral cavity. In contrast, within the first min mark, 5% and 2.5% microstirring pills demonstrated 84% and 56% release respectively, compared to an unobservable quantity found with the control pills (Fig. S5B†). Similarly, increasing the propranolol loading amount in the microstirring pills by ∼6.5 times, had a negligible impact on its full dissolution performance, while initially reducing its release by 1.5 times at first min mark (Fig. S5C and D†). Thus, the enhanced release efficiency of the epinephrine and propranolol microstirring pills further highlights the versatile nature of the microrobotics pill platform for a myriad of clinical indications in which rapid drug delivery is essential. Our *in vitro* results also indicate that both drugs remained stable throughout the reaction process, with no evidence of degradation or interaction with the pill components under the mildly acidic conditions used.

**Fig. 4:**
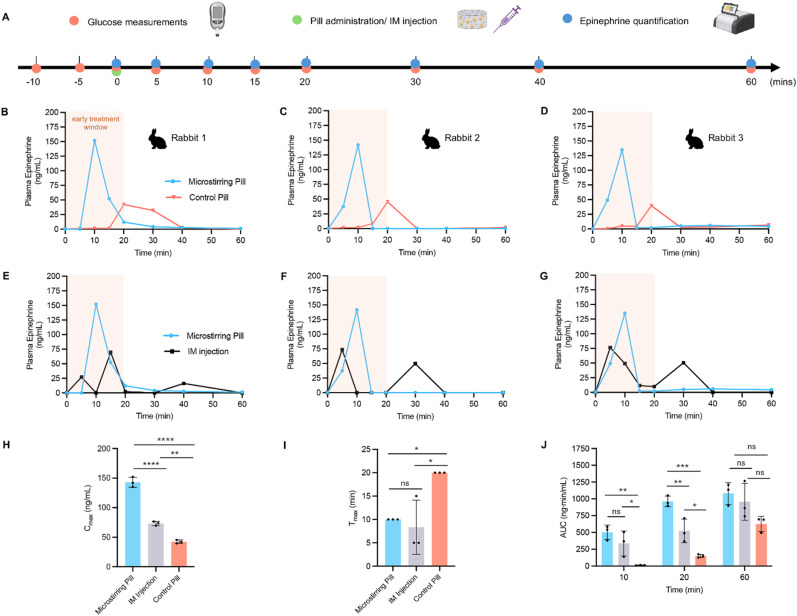
*In vivo* pharmacokinetic study of sublingual epinephrine pills and IM injection in a rabbit model. (A) Timeline of treatment including glucose measurements, pill administration or IM injection and subsequent blood collections for epinephrine quantification. (B–G) Plasma concentration profiles of epinephrine in three rabbits administered microstirring pills, control pills, and intramuscular (IM) injection. Early treatment window to treat anaphylaxis highlighted in pink. (H) Comparison of average peak plasma concentration (*C*_max_) and (I) time to reach peak plasma concentration (*T*_max_) for microstirring pill, control pills, and IM injection. (J) Area under the curve (AUC) analysis at various time points (10, 20, and 60 min) for all three groups. Statistical significance was calculated by one-way ANOVA with post-hoc Tukey's test.

After confirming that the microstirring pills provided faster release of epinephrine than a conventional pill, an *in vivo* study was conducted to investigate whether this effect translates to accelerated systemic absorption in an animal model. New Zealand White rabbits were selected as an ideal animal model due to their adequately sized sublingual space large enough for pill administration, natural resistance to epinephrine and, similar to humans, non-keratinized sublingual mucosa, making them well-suited for evaluating the pharmacokinetics of epinephrine delivery.^33^ In our study, we focused specifically on pharmacokinetic parameters (plasma epinephrine concentrations) over time, which are independent of downstream physiological (pharmacodynamic) effects. Pills were prepared with 10 mg epinephrine loading and administered on the ventral surface of the tongue in the sublingual space. Microstirring pills were prepared with 2.5% microstirrer loading and a separate control pill group was made without microstirrers. Blood glucose measurements were made with a commercial glucometer and blood samples collected at various time points for 1 hour to quantify plasma epinephrine levels using a commercial ELISA kit (Fig. 4A).

As shown in Fig. 4B–D, the microstirring pill consistently reached peak epinephrine levels by 10 min with a mean peak plasma level of 143.0 ± 8.6 ng mL^−1^, compared to a delayed response observed with the control pill reaching 42.4 ± 2.9 ng mL^−1^, 20 min after administration. This demonstrated a 2-fold quicker response time and a 3.4-time enhanced absorption profile with the incorporation of the microstirrers into a conventional pill. Correspondingly, an increasing trend in plasma glucose concentration followed the rise in epinephrine levels observed in the microstirring pill compared to the control pill (Fig. S6A†).

Next, we investigated whether a sublingual microstirring pill could provide a competitive absorption profile compared to an intramuscular (IM) injection of epinephrine, the current gold standard treatment for anaphylaxis. A separate group consisting of 0.3 mg IM injection of epinephrine was selected for administration, corresponding to the standard adult dose found in commercial epinephrine autoinjectors (Fig. 4E–G).^34^ Interestingly, the IM injection group produced two plasma peaks, attributed to the local vascular constriction effect of epinephrine, transiently reducing local epinephrine absorption. As shown in Fig. 4E–G and H, the epinephrine IM injection reached a mean peak plasma concentration of 73.2 ± 3.5 ng mL^−1^, approximately half the peak plasma levels obtained with the microstirrer pill reaching statistical significance (*p* < 0.0001, *α* = 0.05). A similar trend was observed in plasma glucose response, with a higher trend associated with the microstirring pill compared to the IM injection (Fig. S6B†). However, while the IM injection was found to have a variable, yet lower trend in mean *T*_max_ of 8.33 ± 5.8 min (Fig. 4I), no significant difference was observed when compared to the microstirring pill (*p* = 0.819, *α* = 0.05). It is important to note that the penetration depth of IM injections, which can vary due to intrinsic differences in tissue thickness, adiposity, and muscle size, as well as human error, can produce inconsistent plasma epinephrine levels. In contrast, our robotic pill demonstrates the ability to deliver epinephrine quickly, reliably, and with significantly lower variability. This innovative approach holds great promise for the precise delivery of drugs like epinephrine in acute medical conditions, which require a narrow therapeutic window to receive optimal treatment effectively and improve likelihood of patient recovery.^35^

To determine whether our sublingual microstirring platform could potentially be suitable to treat anaphylaxis, we compared its area under the curve (AUC) with that of the epinephrine IM injection across the first 10 and 20 min (Fig. 4J). Anaphylaxis can be fatal if left untreated and delayed epinephrine administration significantly increases the risk of progression to respiratory failure, circulatory collapse from shock, and ultimately cardiac arrest.^36–38^ Importantly, a delay in epinephrine administration is associated with a higher mortality rate. Thus, reaching therapeutic epinephrine levels rapidly within an early treatment window of 10–20 minutes from symptom onset is paramount to improve the chance of survival.^39,40^ Within the first 10 min, the AUC obtained with the microstirring pill demonstrated a 1.5 time higher trend than the standard IM injection (501.6 ± 107.1 ng mL^−1^*vs.* 336.4 ± 187.0 ng mL^−1^) (*p* = 0.306, *α* = 0.05). By the 20 min mark, a 1.8-fold increase was obtained (962.0 ± 79.7 ng mL^−1^*vs.* 523.0 ± 174.7 ng mL^−1^) reaching statistical significance (*p* = 0.0071, *α* = 0.05). Thus, the microstirring pill achieves enhanced epinephrine uptake within the 20 min therapeutic window, demonstrating its superior performance to the gold-standard IM injection. Consequently, the microstirring pill platform provides a competitive therapeutic potential, and if supported by further studies in an anaphylaxis model, may provide a viable alternative to the conventional epinephrine autoinjector.

Similarly, when compared against the control pill, the microstirring pill demonstrated a 39.5-fold greater AUC within 10 min (501.6 ± 107.1 ng mL^−1^*vs.* 12.69 ± 2.7 ng mL^−1^) and a 6.4-fold increase by 20 min (962.0 ± 79.7 ng mL^−1^*vs.* 149.7 ± 24.0 ng mL^−1^). Note that while the control pill reached therapeutic AUC values comparable to IM injection by 60 min, the duration of time required to reach these levels renders it less practical in an acute clinical setting.

## Conclusions

We present the first demonstration of a sublingual microrobotic pill, highlighting its advantage in the swift and efficient delivery of drugs. Owing to its built-in self-mixing capabilities, the microstirrer pill platform provides a robust advantage over static conventional pills by generating a powerful localized fluid mixing that overcomes the diffusion limitations towards enhanced drug transport. Compared to the traditional GI microrobotic pill platform, our sublingual microrobotic pill was carefully designed and optimized to perform safely, swiftly and effectively in both artificial saliva as well as the complex milieu of human saliva, which has its own set of unique challenges, making traditional Mg-based microrobots unsuitable for operation. By harnessing the active propulsion capabilities of Mg microstirrers, the microstirrer pill significantly enhances hydrodynamic flow, accelerates dissolution, and demonstrates faster release of epinephrine and propranolol compared to their static counterparts. Validated by a rabbit model using epinephrine as the model drug, the pharmacokinetic studies confirmed that our sublingual microstirring pill has superior delivery speed and higher bioavailability compared to a conventional pill, and demonstrates a competitive absorption profile against intramuscular injection, which is the standard route of treatment for anaphylaxis. Such sublingual robotic pills would critically benefit other acute medical conditions in connection to greatly enhancing the active delivery of the selected therapies requiring rapid therapeutic action. While the sample size in this study was limited to *n* = 3 per group, the pharmacokinetic profiles were consistent and statistically significant across animals. Further studies involving larger cohorts and disease-specific models are planned to support the translational advancement of the microrobotic sublingual platform toward clinical application. More detailed biosafety evaluations, including histological analysis of local tissue response, will also be valuable to confirm the biocompatibility of the microrobotic propulsion system and support future clinical translation. Beyond its effectiveness in emergency situations, this user-friendly, non-invasive, and cost-efficient platform can be extended to non-emergency applications. Compared with intravenous or intramuscular routes of delivery that require medically trained personnel, this platform may address the critical unmet medical needs in remote and underserved communities, while presenting a significant opportunity to reduce healthcare costs. Moreover, this platform can address the unique set of challenges associated with traditional oral therapies, particularly for patients that require rapid therapeutic action or those suffering from dysphagia who deserve an innovative, non-invasive platform that provide ease in self-administered medication. Thus, this innovative sublingual microrobotic pill technology represents a transformative solution in drug delivery, offering faster therapeutic onset while improving accessibility for wide range of needs.

## Author contributions

Nelly Askarinam: investigation, data curation, formal analysis, visualization, writing – original draft. Chuanrui Chen: investigation, data curation, formal analysis, visualization, writing – original draft. Vivian Vo: investigation, data curation, writing – original draft. Michael Casares: investigation, data curation, formal analysis. Kyra Wu: investigation, data curation, visualization. Ethan Shen: investigation, data curation. Majd Iskandarani: investigation, data curation. Víctor de la Asunción-Nadal: investigation, data curation, visualization, writing – original draft. An-Yi Chang: investigation, data curation. Tomohiro Yamamoto: investigation, data curation. Janna Sofia Sage-Sepulveda: investigation, data curation. Baha Öndeş: investigation, data curation. Zhenning Zhou: investigation, data curation. Zike Yan: investigation, data curation, visualization. Jesse Qualliotine: conceptualization, investigation, formal analysis, visualization, writing – original draft, funding acquisition. Joseph Wang: conceptualization, investigation, formal analysis, visualization, writing – original draft, funding acquisition.

## Conflicts of interest

There are no conflicts to declare.

## Supplementary Material

NA-007-D5NA00313J-s001

NA-007-D5NA00313J-s002

NA-007-D5NA00313J-s003

NA-007-D5NA00313J-s004

## Data Availability

The data supporting this article have been included as part of the ESI.†

## References

[cit1] Budnitz D. S., Lovegrove M. C., Shehab N., Richards C. L. (2011). N. Engl. J. Med..

[cit2] Alqahtani M. S., Kazi M., Alsenaidy M. A., Ahmad M. Z. (2021). Front. Pharmacol..

[cit3] Hatefi A., Amsden B. (2002). J. Control. Release.

[cit4] Jain K. K. (2008). Drug Deliv. Syst..

[cit5] Madibone M. N., Gaikwad S. S., Nikam V. K. (2018). Appl. Clin. Res. Clin. Trials. Regul. Aff..

[cit6] Hua S. (2019). Front. Pharmacol..

[cit7] Narang N., Sharma J. (2011). Int. J. Pharm. Pharm. Sci..

[cit8] Mašek J., Lubasova D., Lukáč R., Turanek-Knotigova P., Kulich P., Plockova J., Mašková E., Prochazka L., Koudelka Š., Sasithorn N. (2017). J. Control. Release.

[cit9] Kraan H., Vrieling H., Czerkinsky C., Jiskoot W., Kersten G., Amorij J.-P. (2014). Vaccine.

[cit10] Takx R. A., Suchá D., Park J., Leiner T., Hoffmann U. (2015). Eur. Radiol..

[cit11] Lavonas E. J., Severtson S. G., Martinez E. M., Bucher-Bartelson B., Le Lait M.-C., Green J. L., Murrelle L. E., Cicero T. J., Kurtz S. P., Rosenblum A. (2014). J. Subst. Abuse Treat..

[cit12] Fu Y., Wang A., Tang R., Li S., Tian X., Xia X., Ren J., Yang S., Chen R., Zhu S. (2024). JAMA Neurol..

[cit13] Simó C., Serra-Casablancas M., Hortelao A. C., Di Carlo V., Guallar-Garrido S., Plaza-García S., Rabanal R. M., Ramos-Cabrer P., Yagüe B., Aguado L., Bardia L., Tosi S., Gómez-Vallejo V., Martín A., Patiño T., Julián E., Colombelli J., Llop J., Sánchez S. (2024). Nat. Nanotechnol..

[cit14] Li J., Esteban-Fernández de Ávila B., Gao W., Zhang L., Wang J. (2017). Sci. Robot..

[cit15] Kim M., Nicholas J. D., Puigmartí-Luis J., Nelson B. J., Pané S. (2025). Annu. Rev. Control Robot. Auton. Syst..

[cit16] Sánchez S., Soler L., Katuri J. (2015). Angew. Chem., Int. Ed..

[cit17] Xu T., Gao W., Xu L. P., Zhang X., Wang S. (2017). Adv. Mater..

[cit18] Wang H., Pumera M. (2015). Chem. Rev..

[cit19] Zhang F., Li Z., Chen C., Luan H., Fang R. H., Zhang L., Wang J. (2024). Adv. Mater..

[cit20] Gao W., Wang J. (2014). Nanoscale.

[cit21] Xu H., Medina-Sánchez M., Magdanz V., Schwarz L., Hebenstreit F., Schmidt O. G. (2018). ACS Nano.

[cit22] Karshalev E., Esteban-Fernández de Ávila B., Beltran-Gastelum M., Angsantikul P., Tang S., Mundaca-Uribe R., Zhang F., Zhao J., Zhang L., Wang J. (2018). ACS Nano.

[cit23] Mundaca-Uribe R., Askarinam N., Fang R. H., Zhang L., Wang J. (2024). Nat. Biomed. Eng..

[cit24] Mundaca-Uribe R., Holay M., Abbas A., Askarinam N., Sage-Sepulveda J. S., Kubiatowicz L., Fang R. H., Zhang L., Wang J. (2023). ACS Nano.

[cit25] Mundaca-Uribe R. (2021). et al.. Adv. Sci..

[cit26] Forssell H. (1988). Scand. J. Gastroenterol..

[cit27] Smith J. and Hong-Shum L., Food Additives Data Book, Wiley Online Library, 2003, 56, pp. 76-79

[cit28] Rawas-Qalaji M., Simons F., Simons K. (2006). J. Allergy Clin. Immunol..

[cit29] Kim J. K. (2015). et al.. Biosens. Bioelectron..

[cit30] Lagerlof F., Dawes C. (1984). J. Dent. Res..

[cit31] Goswami T. (2008). et al.. Crit. Rev. Ther. Drug Carrier Syst..

[cit32] Wang Y. (2013). et al.. AAPS J..

[cit33] Rachid O. (2018). et al.. Pharmaceutics.

[cit34] Edwards E. S. (2013). et al.. Ann. Allergy Asthma Immunol..

[cit35] Kemp S. F., Lockey R. F., Simons F. E. R. (2008). World Allergy Organ. J..

[cit36] Robinson M., Greenhawt M., Stukus D. R. (2017). Ann. Allergy Asthma Immunol..

[cit37] Fleming J. T., Clark S., Camargo C. A., Rudders S. A. (2015). J. Allergy Clin. Immunol. Pract..

[cit38] Sicherer S. H., Simons F. E. R. (2017). Pediatrics.

[cit39] Pumphrey R. S. (2003). J. Allergy Clin. Immunol..

[cit40] Pumphrey R. (2000). Clin. Exp. Allergy.

