# Supplementary material for: Sublingual microrobotic pills for rapid and efficient drug delivery
Source: Nanoscale Adv. 2025 Jun 23;7(15):4730–9. doi: 10.1039/d5na00313j (PMC12199271; doi:10.1039/d5na00313j)
Supplement: NA-007-D5NA00313J-s004 [file NA-007-D5NA00313J-s004.pdf]

Supplementary Information (SI) for Nanoscale Advances.
This journal is © The Royal Society of Chemistry 2025

Skip the Needle: Sublingual Microrobotic Pills for Rapid and Efficient Drug Delivery

Nelly Askarinam[a†], Chuanrui Chen[a†], Vivian Vo[d], Michael Casares[a], Kyra Wu[a], Ethan Shen[a], Majd Iskandarani[a], Víctor de la Asunción-Nadal[a], An-Yi Chang[a], Tomohiro Yamamoto[a], Janna Sofia Sage-Sepulveda[a], Baha Öndeş[a], Zhenning Zhou[a], Zike Yan[a], Joseph Wang[a]*, Jesse Qualliotine[b,c]*

a, Aiiso Yufeng Li Family Department of Chemical and Nano Engineering, University of California San Diego, La Jolla, California 92093, United States

b, Moores Cancer Center, University of California, San Diego, La Jolla, CA, USA

c, Department of Otolaryngology - Head and Neck Surgery, University of California, San Diego, La Jolla, CA, USA

d, University of California San Diego, School of Medicine, La Jolla, California 92093, United States

*Corresponding Authors:

Joseph Wang, Aiiso Yufeng Li Family Department of Chemical and Nano Engineering, University of California San Diego, La Jolla, California 92093, United States. Email: josephwang@ucsd.edu

Jesse R. Qualliotine, Department of Otolaryngology - Head and Neck Surgery, University of California San Diego, La Jolla, CA, 92093, USA. Email: jqualliotine@health.ucsd.edu

[†] *These authors contributed equally*

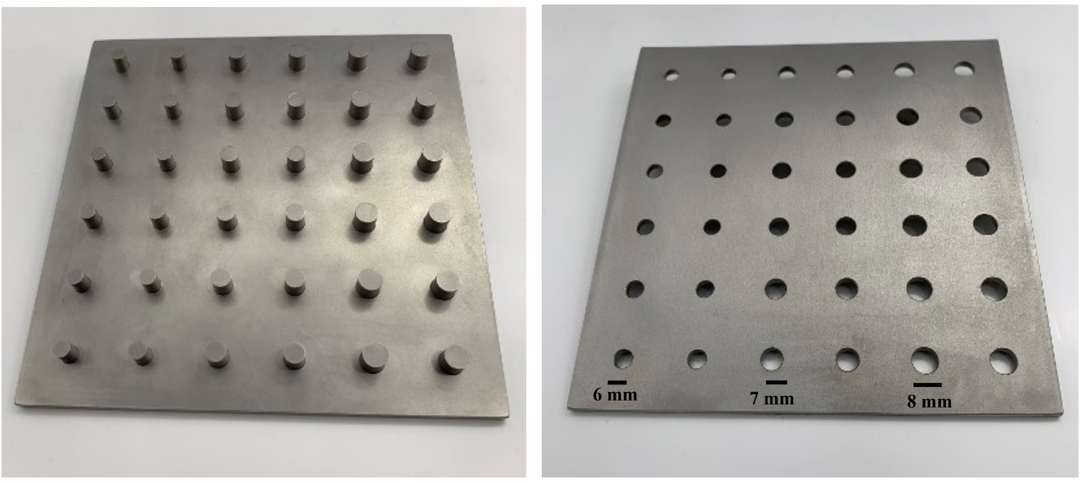

Figure S1. Photograph images of stainless steel 2-part 6×6 array rabbit pill molds consisting of a PEG plate imaged on left and pill mold on right. Pill mold holes are sized with 6 mm, 7 mm and 8 mm diameters and 3 mm height respectively, each arranged in a 2×6 array.

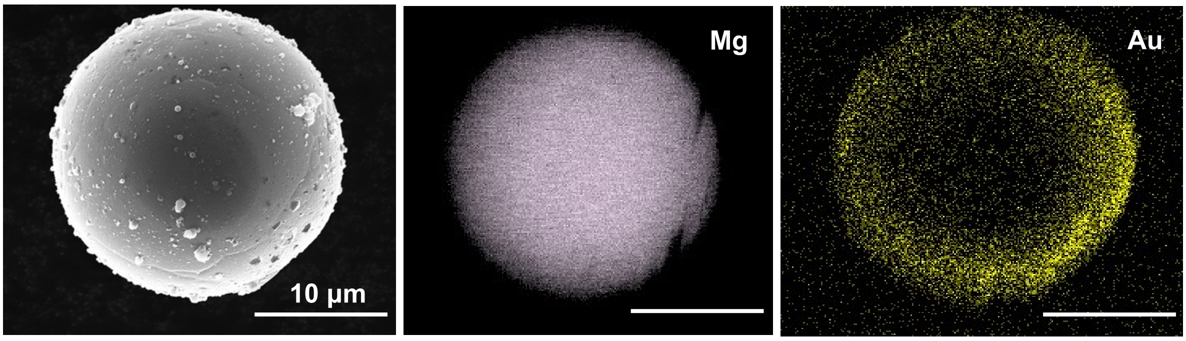

Figure S2. SEM image of a microstirrer composed of outer layer of Au shell shown in light grey and an exposed inner Mg core shaded in dark grey shown on left. Energy-dispersive X-ray spectroscopy (EDX) images of elemental composition of inner Mg core (middle) and outer Au layer (right).

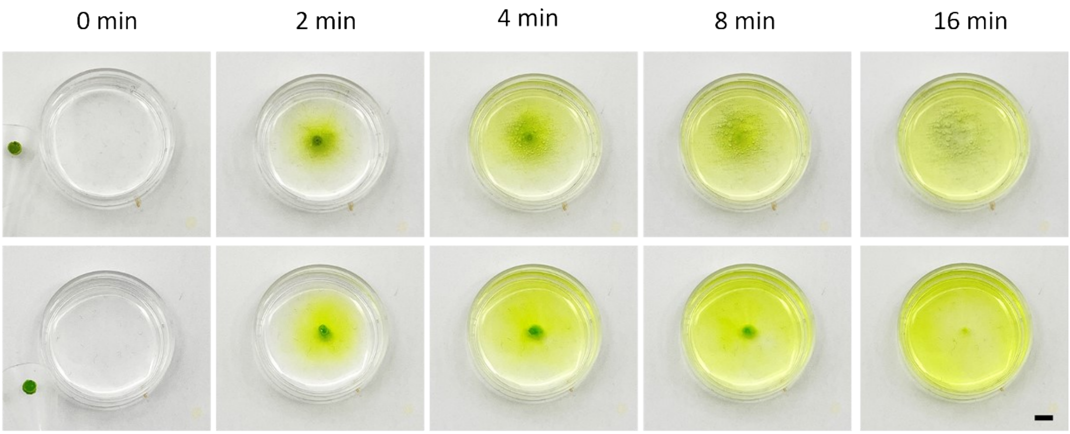

Figure S3. Representative dissolution process of a microstirring pill (loaded with 1.5 wt% Mg/Au microstirrers, upper panel) and a control pill (lower panel) in artificial saliva. Both pills were loaded with green dye to visualize the dissolution process.

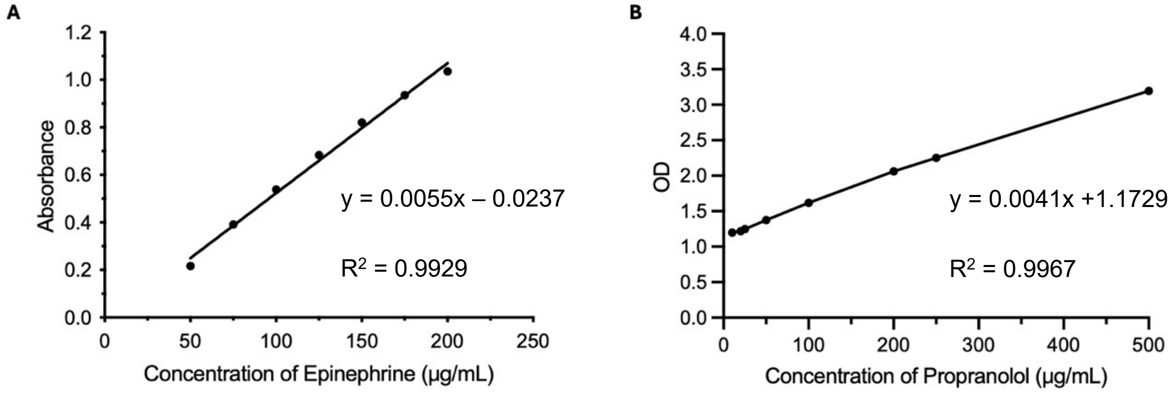

Figure S4. Epinephrine (A) and propranolol (B) calibration curve.

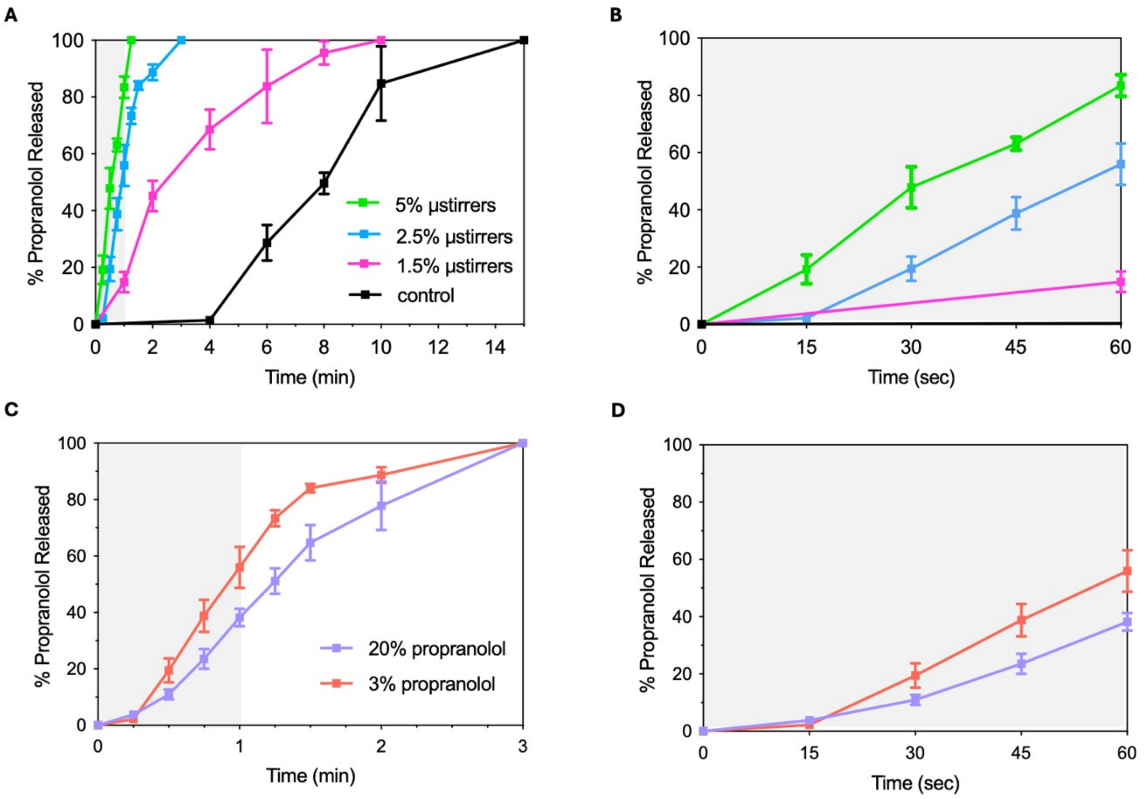

Figure S5. In vitro release kinetics of propranolol from sublingual microstirring and control pills. (A) Kinetic study of propranolol release from microstirring pills with varying loading amounts of microstirrers vs control pill across 15 mins and (B) at first min mark with 3% propranolol loading. (C) Kinetic study of propranolol release from microstirrer pills with various loading amounts of propranolol and 2.5% microstirrer across 3 mins and (D) at the first min mark.

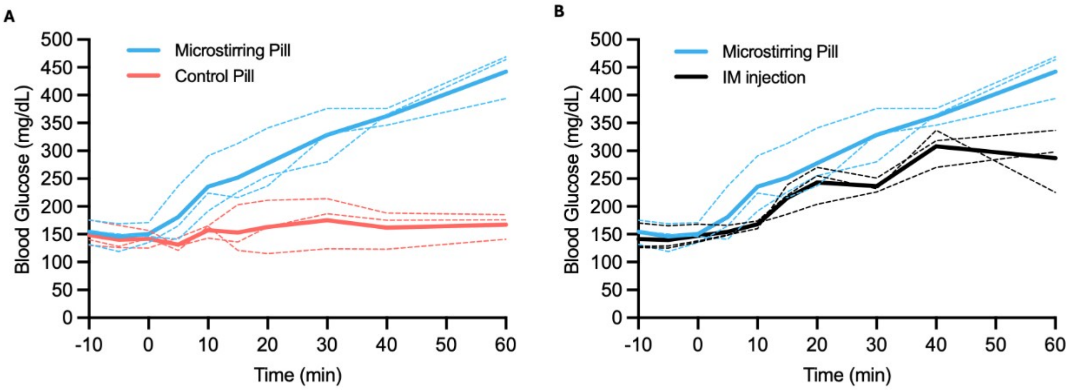

Figure S6. Plasma glucose response shown from sublingual microstirring pill compared to control pill

(A) or IM injection (B) (n = 3). Each replicate is displayed as dotted lines and mean plasma glucose level as solid lines.
